# Association of Medicaid Healthy Behavior Incentive Programs With Smoking Cessation, Weight Loss, and Annual Preventive Health Visits

**DOI:** 10.1001/jamanetworkopen.2018.6185

**Published:** 2018-12-28

**Authors:** Sarah W. Huf, Kevin G. Volpp, David A. Asch, Elizabeth Bair, Atheendar Venkataramani

**Affiliations:** 1 Department of Surgery and Cancer, Imperial College London, St Mary’s Hospital, London, United Kingdom; 2 Center for Health Care Innovation, Perelman Center for Advanced Medicine, Philadelphia, Pennsylvania; 3 Division of Health Care Policy, University of Pennsylvania, Philadelphia

## Abstract

**Importance:**

Several states have implemented Healthy Behavior Incentive Programs (HBIPs) in Medicaid through Section 1115 demonstration waivers. These programs use financial incentives to encourage positive behavior changes, such as greater use of preventive services, smoking cessation, and weight loss.

**Objective:**

To test for an association between the introduction of HBIPs and the rates of smoking cessation, weight loss, and annual preventive health visits in states that have adopted behavior-specific programs compared with states that have not.

**Design, Setting, and Participants:**

A cohort study using a difference-in-differences analysis of the 2011-2016 Behavioral Risk Factor Surveillance Survey Interview Results data, adjusting for demographic conditions, state unemployment rates, state Medicaid expansion, national secular trends, and time invariant state-specific factors, was conducted. Two sets of participants were considered: adults aged 18 to 64 years who had a reported annual household income of less than $25 000 (n = 442 089) or adults aged 18 to 64 years who had completed high school education or less (n = 676 883).

**Exposures:**

Changes in health behavior outcomes in 4 states (Florida, Indiana, Iowa, and Michigan) that implemented behavior-specific HBIPs targeting smoking, obesity, and annual health checkups through a Section 1115 waiver, against changes in control states, including Washington, DC, that did not introduce an HBIP (n = 44).

**Main Outcomes and Measures:**

Rate of smoking, obesity, and attendance at annual preventive health visits.

**Results:**

Of Behavioral Risk Factor Surveillance Service respondents used for the less than $25 000 annual household income cohort (n = 442 089), the mean (SD) age was 43.1 (0.8) years, and the mean (SD) percentage of women was 58.4% (2.5%). For the cohort of high school education or less (n = 676 883) population, the mean (SD) age was 41.6 (1.1) years, and the mean (SD) percentage of women was 46.6% (0.9%). During a 2-year period after implementation, there were no improvements in smoking and obesity in individuals with a household income of less than $25 000 (2.49 percentage points, 95% CI, 1.75-3.23 percentage points; *P* < .001 and −1.94 percentage points, 95% CI, −4.42 to 0.55 percentage points; *P* = .12, respectively) as well as in the population holding a high school education or less (1.74 percentage points, 95% CI, 0.64-2.85 percentage points; *P* = .003 and −0.73 percentage points, 95% CI, −1.84 to 0.38 percentage points; *P* = .19). An association was noted between an increase in preventive health visit rates among states adopting behavior-specific HBIPs relative to control states in the less than $25 000 household income population (3.89 percentage points, 95% CI, 2.64-5.14 percentage points; *P* < .001). However, these associations were substantively small and not robust across the high school education or less population (1.8 percentage points, 95% CI, −0.12 to 3.71 percentage points; *P* = .07).

**Conclusions and Relevance:**

Early postimplementation assessment may indicate that HBIPs were not associated with substantive improvements in incentivized healthy behaviors among populations likely to be Medicaid beneficiaries. The value, format, and timing of the incentive, complexity in delivery, and lack of awareness of incentives among target beneficiaries and clinicians may limit the usefulness of programs even over a longer follow-up period.

## Key Points

**Question:**

Are Medicaid Healthy Behavior Incentive Programs associated with improvements in healthy behaviors and population health?

**Findings:**

In this cohort study based on data from 442 089 low-income individuals and 676 883 individuals with a high school education or less, there were no significant improvements in incentivized behaviors, such as smoking cessation or weight loss, in either group. An association was found between an increased rate of annual health checkups, but the association was not robust across models.

**Meaning:**

Medicaid Healthy Behavior Incentive Programs appear to have had little, if any, positive association with key health behaviors in the first 2 years of implementation.

## Teaser

This cohort study evaluates the association between the introduction of Medicaid Healthy Behavior Incentive Programs and the rates of smoking cessation, weight loss, and annual preventive health visits.

## Introduction

Health risk behaviors, such as smoking, consuming a poor diet, engaging in limited physical activity, and not participating in health screening, can increase the risk of avoidable premature morbidity and mortality.^1,2^ These behaviors have proved difficult to modify.^3,4^ Recent research from the field of behavioral economics suggests that financial incentives may be effective in achieving smoking cessation,^5,6^ weight loss,^7^ increased exercise,^8,9,10^ and medication adherence.^11^ Consequently, there is growing interest in implementing these initiatives at the population level.

Rates of unhealthy behaviors, such as smoking, are higher among Medicaid beneficiaries than in the privately insured population.^12^ There has been mounting interest among states—regardless of whether they expanded access to Medicaid as part of the Affordable Care Act—to improve population health among Medicaid beneficiaries by directly incentivizing key healthy behaviors using financial incentives. Seven states (Arizona, Florida, Iowa, Indiana, Michigan, New Mexico, and Kentucky) have received a waiver to enact Healthy Behavior Incentive Programs (HBIPs). Of these states, Florida, Michigan, Iowa, and New Mexico have had HBIPs in place for more than 4 years, while Indiana’s has been in place for more than 3 years and Arizona’s HBIPs have been in place for more than 2 years. Kentucky’s HBIP was approved in early 2018 but suspended by a court ruling and currently remains under review; Wisconsin currently holds a pending application.^13,14^ Examples of proposed or enacted incentives across these programs include reductions in or waivers of premiums, rollover of saved funds in health accounts, or gift cards.^15^ However, the effects of these HBIPs on targeted health behaviors in these programs is not known.

The objective of this study was to assess whether there is an association between the introduction of state-level Medicaid behavior-specific HBIPs and changes in the incentivized health behaviors, specifically annual checkups, smoking status, and body mass index (BMI).

## Methods

### Study Design

We used a difference-in-differences research design to compare changes in the rates of 3 predetermined healthy behavior outcomes—cigarette smoking, obesity (as defined by BMI >30 [calculated as weight in kilograms divided by height in meters squared]), and annual health checkups—before and after the implementation of HBIPs, which specifically addressed these behaviors through Section 1115 waivers, against the same changes in states that did not implement an HBIP waiver (control states). We identified states with these behavior-specific incentives implemented at a time that allowed for at least 2 years of postpolicy follow-up, including the year of implementation as of 2016. Four states met those criteria: Florida, Michigan, Indiana, and Iowa. These states targeted smoking cessation (Florida, Michigan), weight loss (Florida, Michigan), and/or attendance at annual health checkups (all 4 states). Washington, DC, was included in the control states. States whose programs had been in place for less than 2 years (Arizona), had not yet implemented their program (Kentucky), or had implemented programs incentivizing behaviors other than the preselected outcomes (New Mexico) were not included in either the intervention or control group.

Per the University of Pennsylvania Institutional Review Board's policy guidance on projects using publicly available data, no institutional review board protocol review was required. This study followed the Strengthening the Reporting of Observational Studies in Epidemiology (STROBE) reporting guideline.^16^

### Outcomes and Covariates

We used data from 2011-2016 waves of the US Behavioral Risk Factor Surveillance Survey (BRFSS), an annual, state, and nationally representative telephone-based survey of the general, noninstitutionalized US population conducted by the Centers for Disease Control and Prevention. We restricted our sample to 2 populations of individuals who *ex ante* could be most likely to access Medicaid. Specifically, we selected individuals between the ages of 18 and 64 years who reported an annual household income below $25 000 per year and individuals of the same age range who completed high school or less education.

Because 12% of the sample reported that they did not know their household income or refused to answer the question, and because selecting a population based on income alone may be problematic as participation in Medicaid may affect employment and financial outcomes, we followed prior work by also estimating our models among survey participants of the same age range reporting high school education or less as an additional check.^17^

Using BRFSS population weights, we constructed our primary outcomes: state-year annual rates of smoking, obesity (BMI >30), and having had a preventive health checkup in the year before the survey among the sample populations. We additionally created measures for key covariates, specifically age, sex, race/ethnicity, employment status, household below the federal poverty line (FPL), and Affordable Care Act Medicaid expansion, using data from the American Community Survey.^18^

### Statistical Analysis

We conducted difference-in-differences analyses for each primary outcome. To do so, we fitted a linear regression model that included an interaction term for behavior-specific HBIP waiver implementation and whether the survey year occurred after HBIP waiver implementation along with state- and year-fixed outcomes, which adjust for the main effects for whether a state implemented a behavior-specific HBIP and whether the survey year occurred after implementation.^19^ The coefficient on the interaction term represents the difference-in-differences estimate: it measures the changes in healthy behavior rates in HBIP implementation states before and after HBIP implementation relative to the same change in states that did not implement an HBIP waiver (specific to smoking cessation, obesity, or attendance at annual health checkups) over that time period. The state and year fixed effects adjust for time-invariant state characteristics and national trends in the outcomes of interest, respectively. We additionally adjusted for state-year demographic characteristics (age, sex, and racial/ethnic distribution) and socioeconomic factors (unemployment and household income below the FPL).

We also adjusted for whether states expanded Medicaid as part of the Affordable Care Act, using a binary indicator equal to 1 if a Medicaid expansion was operative in a particular state-year. Among treated states, Michigan, Indiana, and Iowa expanded their Medicaid program in 2014, but Florida did not. By adjusting for this binary indicator, the difference-in-differences estimates capture associations between HBIPs and the outcomes net of any results of Medicaid expansions.^20,21,22^

For all analyses, SEs were clustered at the state level to account for serial correlation in the outcomes. A 2-tailed *P* value <.05 was considered statistically significant.^23^ Stata, version 15.1 (StataCorp), was used for all analyses.

### Results

The data of 408 847 American Community Survey respondents and 142 088 BRFSS respondents from 2011 to 2012 were used to create state-year level measures of the preimplementation baseline demographics socioeconomic characteristics and baseline healthy behavior rates. Within the less than $25 000 household income population, the mean (SD) age was 43.1 (0.8) years, and the mean (SD) percentage of women in the population was 58.4% (2.5%). Overall, with regard to the mean (SD) percentage of participants, 65.5% (10.8%) were white, 21.4% (11.9%) were black, and 13.5% (8.5%) were of other racial/ethnic minority groups, 18.5% (14.0%) were Hispanic, 51.3% (4.2%) were employed, and 59.0% (2.7%) fell below the FPL. Within the high school education or less population, the mean (SD) age was 41.6 (1.1), and the mean (SD) percentage of women was 46.9% (0.9%). Overall, with regard to the mean (SD) percentage of participants, 70.7% (10.7%) were white, 15.1% (9.6%) were black, and 14.2% (10.2%) were of other racial/ethnic minority groups, 22.7% (17.8%) were Hispanic, 60.1% (3.6%) were employed, and 30.9% (3.3%) fell below the FPL. The preimplementation baseline demographics and socioeconomic characteristics for the 4 HBIP waiver–approved states and states without such waivers are reported in Table 1.

**Table 1.** Baseline Characteristics and Healthy Behavior Rates Before HBIP Waiver Adoption (2011-2012)

Baseline Characteristics (n = 408 847)^a^	Mean % (SD)	
	Behavior-Specific HBIP Waiver-Approved States (n = 4)	Non-HBIP Waiver-Approved States (n = 44)
Age, y		
18-34	35.2 (4.8)	36.5 (3.5)
35-54	41.7 (3.3)	41.5 (2.7)
55-64	23.0 (1.9)	21.9 (1.8)
Women	57.7 (0.9)	58.5 (2.6)
Race/ethnicity		
White	71.3 (5.6)	64.5 (11.1)
Black	21.4 (5.6)	21.4 (12.7)
Other	7.3 (0.7)	14.7 (8.9)
Hispanic	15.7 (10.4)	18.9 (1.5)
Employed	51.4 (3.5)	51.3 (4.3)
Below FPL	57.7 (2.6)	59.2 (2.7)
Healthy behavior rates (n = 142 088)^b^		
Smoking cessation^c^	32.9 (6.3)	33.0 (7.7)
BMI >30^c^	31.5 (3.0)	30.6 (3.9)
Annual health checkup^d^	52.9 (3.0)	56.1 (7.3)

Abbreviations: BMI, body mass index (calculated as weight in kilograms divided by height in meters squared); FPL, federal poverty line; HBIP, Healthy Behavior Incentive Program.

^a^ Demographic, employment, and FPL data were sourced from the American Community Survey.

^b^ Healthy behavior rate data were sourced from the Behavioral Risk Factor Surveillance System survey data from 2011-2012, before the first HBIP waiver was approved.

^c^ Determined in Florida and Michigan.

^d^ Determined in Florida, Indiana, Iowa, and Michigan.

For the primary outcomes, data of 442 089 and 676 883 BRFSS respondents between 2011 and 2016 were used for the less than $25 000 annual household income and high school education or less populations, respectively. The data of 1 133 289 and 5 026 754 American Community Survey respondents between 2011-2016 were used for the less than $25 000 annual household income and high school education or less populations, respectively.

The Figure presents changes in each the primary outcomes over the study period. The arrows in the figure indicate the year of HBIP-waiver implementation. Figure, A shows the mean annual rate of current smokers in non–HBIP-waiver states compared with Michigan and Florida, which show similar preimplementation rates with a gradual downward trend over the 5-year period. Figure, B presents the rate of obesity (BMI >30), which demonstrates that both implementation states tracked close to the non–HBIP-waiver control group both before and after their implementation years. Figure, C represents the time change in annual health checkup rates in the control group compared with Michigan, Florida, Indiana, and Iowa. Herein, all 4 implementation states started with similar baseline rates of annual health checkups compared with the control group and continuing along a trend similar to that of the control group after HBIP implementation.

**Figure.**
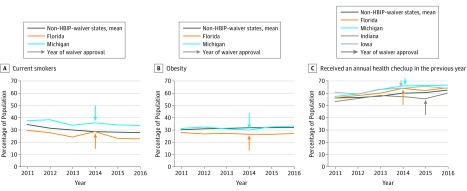
Changes in Healthy Behavior Indicators Before and After Implementation of Behavior-Specific Healthy Behavior Incentive Program (HBIP) Waivers A, Percentage of current smokers. B, Obesity (percentage of population with body mass index >30 [calculated as weight in kilograms divided by height in meters squared]). C, Received an annual health checkup in the previous year. Changes in healthy behavior indicators waiver-approved states (Florida, Michigan, Indiana, and Iowa) and the mean of non–HBIP-waiver states, using Behavioral Risk Factor Surveillance System–weighted and state-population–weighted data analyses, before and after HBIP waiver implementation. Arrows indicate the year of waiver approval of individual states.

Consistent with the Figure, there was no association between the introduction of HBIP programs and an improvement in smoking (2.49 percentage points; 95% CI, 1.75-3.23 percentage points; *P* < .001) or obesity (−1.94 percentage points; 95% CI, −4.42 to 0.55 percentage points; *P* = .12) rates in the difference-in-differences estimates comparing HBIP states (n = 2) with the control states (n = 44) in the less than $25 000 household income population, as well as in the population receiving a high school education or less (1.74 percentage points; 95% CI, 0.64-2.85 percentage points; *P* = .003 and −0.73 percentage points; 95% CI, −1.84 to 0.38 percentage points; *P* = .19, respectively) (Table 2).

**Table 2.** Difference-in-Differences Regression Analysis for HBIP-Waiver States vs Non–HBIP-Waiver States

Incentivized Healthy Behavior	HBIP-Waiver States vs Non–HBIP-Waiver States^a^			
	Household Income <$25 000		Education ≤High School	
	% Point Change (95% CI)^b^	*P* Value	% Point Change (95% CI)^b^	*P* Value
Smoking^c^	2.49 (1.75 to 3.23)	<.001	1.74 (0.64 to 2.85)	.003
BMI >30^c^	−1.94 (−4.42 to 0.55)	.12	−0.73 (−1.84 to 0.38)	.19
Annual health checkup^d^	3.89 (2.64 to 5.14)	<.001	1.80 (−0.12 to 3.71)	.07

Abbreviations: BMI, body mass index (calculated as weight in kilograms divided by height in meters squared); HBIP, Healthy Behavior Incentive Program.

^a^ Household income and education levels before and after HBIP-waiver implementation. Implementation occurred in 2014 in Florida, Michigan, and Iowa, and in 2015 in Indiana. comparing states with healthy behavior-specific waivers against states with no Healthy Behavior Incentive Section 1115 waivers (44 states), adjusted for demographics, socioeconomic factors, and the expansion of Medicaid as part of the Affordable Care Act in different states.

^b^ Annual percentage point change for each behavior.

^c^ Determined in Florida and Michigan.

^d^ Determined in Florida, Indiana, Iowa, and Michigan.

Although point estimates for obesity indicated a minor improvement in HBIP-waiver states, estimates were substantively small and not statistically significant. Estimates for the smoking rate suggested a slower decline in smoking in the 2 implementation states than in the control states, although again these estimates were small and their statistical significance varied in the 2 populations analyzed.

Estimates suggested potential increases in annual health checkup rates, although the magnitude and significance varied by study population. In our main sample of individuals reporting annual household incomes less than $25 000, there was an associated statistically significant increase (3.89 percentage points; 95% CI, 2.64-5.14 percentage points; *P* < .001) in checkup rates. However, the estimate using the sample of individuals with high school education or less was smaller and not statistically significant (1.8 percentage points; 95% CI, −0.12 to 3.71 percentage points; *P* = .07) (Table 2). Estimates for covariates for all regressions are presented in the eTable in the Supplement.

## Discussion

In these early results of nationally representative survey data, we found that HBIPs implemented in Indiana, Iowa, Michigan, and Florida were not associated with improved rates of smoking cessation or weight loss in a 2-year follow-up period among individuals most likely to access the Medicaid program. The HBIPs may have been associated with a small increase in annual checkup rates, although the estimates were not robust across the 2 populations analyzed.

Although the lack of association could be the result of expected lags in treatment outcomes that may not manifest over a short follow-up period, these results provide some evidence that the approaches being adopted by states to improve health behaviors among Medicaid beneficiaries are showing little substantive change in behavior. There are a number of elements that suggest that null effects could persist.

The first of these elements is that the current design of HBIP incentives may be limiting their potential success. First, most incentives provided by the HBIPs are smaller in value compared with incentives tested in other research studies that were successful^5,6,7,8,9,10,11^ and may have been too small to be salient to health care users. For example, in Florida’s Managed Medical Assistance waiver, agreeing to address a healthy behavior, such as smoking cessation, was rewarded with an incentive of between $20 and $50 for single or multiple behavior episodes, respectively,^24^ compared with rewards of up to $800 in clinical trials.^5,6^ In Iowa, participation in a target behavior is rewarded with the waiving of premiums for patients both above and below the FPL, resulting in potential savings of only $10 and $5 per month, respectively.^25^ Recognizing the concern of ineffective low dollar value incentives, the Healthy Indiana Plan 2.0 has applied for a waiver amendment to raise its previous reward dollar limit ($10-$25) to a $200 limit per behavior episode with a $300 annual limit, which was approved in early 2018.^26^

Second, tying rewards to larger streams of funding, such as premiums, can reduce the perception of their size and salience. Third, in Florida, the incentives could be redeemed through gift cards, vouchers, and credits to health accounts or debit cards to purchase approved health-related products, supplies, and services.^24^ Because rewards are valued less when they are chosen by others,^27^ in this case the Medicaid program’s choice of reward rather than cash, the design limits the reward potency.

Fourth, the promised delivery of the rewards was often far in the future in relation to the incentivized behavior. For example, in Michigan, adherence to a specified behavior results in a 50% reduction in premiums the following year for those above the FPL and a $50 gift card for those below the FPL.^28^ In Indiana, patients are allocated a power account and can roll over a portion of unused funds to the following year by participating in a preventive service.^26^ Such delays generally weaken effectiveness.^29,30^ Furthermore, administrative delays in delivering rewards may have also reduced effectiveness, as an evaluation of the Healthy Indiana Plan found that there was often a 4- to 5-month delay in receiving the incentives after completing a target behavior owing to the time required for health care professionals and health plans to settle the payment for the POWER account.^26^ In this setting, promising advances in technology to measure behaviors, such as physical activity, dietary intake, or smoking cessation through, for example, biomarkers, remote sensing devices, or bidirectional communication via text messaging, could in the future provide an accurate and immediate route to track and reward behavior change.^31,32,33^

Fifth, challenges in communicating complex changes to the existing Medicaid rules to the beneficiaries and health care professionals may have led to poor awareness of the program and knowledge of program rules, contributing to reduced adoption. Overall, this lack of understanding resulted in fewer beneficiaries taking advantage of the available incentives. For example, in Iowa, an early postimplementation analysis published just over a year after the waiver was implemented found that more than 90% of IowaCare members were not aware that completing an annual health checkup would result in their contributions ($5-$10 per month) being waived,^25^ resulting in low incentive uptake.^34^ Communication challenges may be exacerbated among the most vulnerable of intended beneficiaries. Evidence from a previous initiative in Florida (the Florida Medicaid Enhanced Benefits Rewards Program) highlighted that racial or ethnic minority groups, non-English speakers, individuals with less than a high school education, or those without primary care clinicians were less likely to be aware of and use the program.^35^ Therefore, reducing the complexity of the incentive programs, as well as dissemination of transparent and easy-to-understand information for beneficiaries and clinicians, may play a key role in the success of such HBIPs on behavior change.

Sixth, the complexity of program design, particularly around beneficiary tracking for premium contributions, behavior tracking, and incentives allocation, may also have reduced effectiveness. Early evidence from Michigan, Iowa, and Indiana has indicated that clinicians and beneficiaries had limited understanding of their state’s HBIP policies.^34,36^

Seventh, the HBIPs examined in this study were not introduced in a vacuum—previous efforts to use healthy behavior incentives within Medicaid through other programs have been adopted without being introduced through waivers. For example, the Medicaid Incentives for Prevention and Chronic Diseases program provided funds to 10 states over 5 years starting in 2011 to target a number of healthy behaviors, including tobacco cessation and weight loss. These programs were concluded in January 2016.^37^ It is therefore possible that the current programs did not result in a difference sufficient to be detectable on top of any effects of previous incentive programs.

### Limitations

This study has several limitations. First, the analysis provides an assessment of a potential association in only the first 2 to 3 years after initiation of HBIPs since these were changes recently implemented. Second, the rates of behavior change were assessed using data from the BRFSS, which relies on self-reported outcomes that can be inaccurate. Some states adopting smoking-specific waivers include biochemical validation of smoking cessation and, thus, the BRFSS self-reported smoking status is not an exact match.^31^ Although no research was found that validates the BRFSS current smoker rate to biochemically validated smoking rates, evidence suggests that the BRFSS performs well in measuring smoking rates at the population level.^38^ Although self-report bias also occurs with obesity, it is unlikely to affect our estimates as the rate of underreporting or overreporting of weight and height is unlikely to vary by state or by year. If individuals were self-reporting lower weights differentially, they would, if anything, be more likely to do so in states with HBIPs that address obesity, moving the estimates away from the null. The lack of an association being found suggests that this bias is unlikely to have affected results.

Third, we could not identify Medicaid beneficiaries directly through the BRFSS for the study period assessed, and instead approximated that population through alternative methods described in the literature.^39^ However, using the population reporting an annual household income of less than $25 000 per year has limitations as Medicaid eligibility criteria vary by household size and composition. Previous research has dealt with this variation by focusing on individuals whose income falls below the 100% FPL.^22^ This method was considered for our study. However, incomplete BRFSS data on household size through 2013, as a result of respondents with only landlines (more than a fifth of the BRFSS population) being asked this question resulted in challenges calculating the FPL status for a large, select group of respondents. Consequently, we did not pursue this approach.

Fourth, it is possible that time-varying factors at the state level, such as other changes to the Medicaid program, may confound our findings. This possibility remains in all observational studies, even after the steps we took to reduce bias, including adjusting for confounders (Medicaid eligibility thresholds and Affordable Care Act Medicaid expansions, state economic conditions), reassessment of effects with different control groups, and specific focus on behaviors incentivized by HBIPs. Fifth, as with all difference-in-differences analyses, estimates may be biased if treated and control states followed different trajectories in the preintervention period, if other events or state policies coincided with treatment, or if outcomes regressed to the mean after treatment implementation. Although these assumptions cannot be directly tested, our analysis of visual trends and stability of substantive findings to changes depending on the population analyzed suggested that bias from these factors was not material. Sixth, in ecologic studies such as this, it is not possible to rule out associations for specific subgroups of patients.

## Conclusions

State Medicaid programs are increasingly adopting personal responsibility–based incentives programs to motivate behavior change among their beneficiaries. Early evidence suggests that, as designed, these programs are not successful. Higher-value incentives, provided at or near the time of completing a healthy behavior, with currencies that are both more salient and more valued by beneficiaries, may be more effective. Simplification of a program’s incentive and reward policy to increase understanding for both the potential incentive beneficiaries and clinicians may improve the usefulness of HBIPs.

## Notes

Supplement. **eTable.** Covariates for the Difference-in-Differences Regression Analysis Presented in Table 2, for the <$25 000 Household Income Population
