# Supplementary material for: Association of Medicaid Healthy Behavior Incentive Programs With Smoking Cessation, Weight Loss, and Annual Preventive Health Visits
Source: JAMA Netw Open. 2018 Dec 28;1(8):e186185. doi: 10.1001/jamanetworkopen.2018.6185 (PMC6324555; doi:10.1001/jamanetworkopen.2018.6185)
Supplement: Supplement. — eTable. Covariates for the Difference-in-Differences Regression Analysis Presented in Table 2, for the <$25 000 Household Income Population [file jamanetwopen-1-e186185-s001.pdf]

Supplementary Online Content

Huf SW, Volpp KG, Asch DA, Bair E, Venkataramani E. Association of Medicaid Healthy Behavior Incentive Programs with smoking cessation, weight loss, and annual preventive health visits. *JAMA Netw Open*. 2018;1(8):e186185. doi:10.1001/jamanetworkopen.2018.6185

eTable. Covariates for the Difference-in-Differences Regression Analysis Presented in Table 2, for the <$25 000 Household Income Population

This supplementary material has been provided by the authors to give readers additional information about their work.

© 2018 Huf SW et al. *JAMA Network Open.*

eTable. Covariates for the Difference-in-Differences Regression Analysis Presented in Table 2, for the <$25 000 Household Income Population.

	Smoking			BMI > 30kg/m2			Annual Check-up		
	β	95% CI	*p*	β	95% CI	*p*	β	95% CI	*p*
**Age, y**									
18-34	(ref)			(ref)			(ref)		
35-54	-0.13	(-0.36, 0.10)	0.28	0.14	(-0.03, 0.31)	0.10	0.13	(-0.17, 0.44)	0.38
54-64	0.22	(-0.16, 0.60)	0.25	0.11	(-0.19, 0.40)	0.47	0.18	(-0.19, 0.56)	0.33
**Female**	0.30	(0.01, 0.58)	0.05	-0.22	(-0.49, 0.06)	0.12	0.25	(-0.02, 0.52)	0.07
**Race**									
White	(ref)			(ref)			(ref)		
Black	0.18	(-0.25, 0.62)	0.40	-0.03	(-0.39, 0.33)	0.87	-0.21	(-0.56, 0.14)	0.23
Other	-0.08	(-0.38, 0.22)	0.60	0.08	(-0.19, 0.35)	0.54	-0.03	(-0.48, 0.42)	0.89
**Hispanic**	-0.02	(-0.36, 0.32)	0.92	-0.28	(-0.61, 0.06)	0.11	-0.12	(-0.63, 0.39)	0.64
**Employed**	0.02	(-0.20, 0.24)	0.84	-0.11	(-0.38, 0.16)	0.41	0.02	(-0.23, 0.27)	0.87
**Household below FPL**	-0.07	(-0.27, 0.12)	0.45	-0.18	(-0.43, 0.08)	0.17	0.05	(-0.20, 0.30)	0.70
**ACA expansion**	0.00	(-0.01, 0.01)	0.68	0.00	(-0.02, 0.01)	0.55	0.01	(0.00, 0.03)	0.05

Notes: Covariates for the difference-in-differences regression analysis of states with behaviour-specific Healthy Behavior Incentive Program waivers vs 44 control states, which do not hold an approved HBIP waiver using the Behavioral Risk Factor Surveillance System and state specific population weighted data in individuals with ≤ $25 000 (Table 2). FPL denotes Federal Poverty Line. *p* denotes p-value.

© 2018 Huf SW et al. *JAMA Network Open.*
